# Habituation based synaptic plasticity and organismic learning in a quantum perovskite

**DOI:** 10.1038/s41467-017-00248-6

**Published:** 2017-08-14

**Authors:** Fan Zuo, Priyadarshini Panda, Michele Kotiuga, Jiarui Li, Mingu Kang, Claudio Mazzoli, Hua Zhou, Andi Barbour, Stuart Wilkins, Badri Narayanan, Mathew Cherukara, Zhen Zhang, Subramanian K. R. S. Sankaranarayanan, Riccardo Comin, Karin M. Rabe, Kaushik Roy, Shriram Ramanathan

**Affiliations:** 1 School of Materials Engineering, Purdue University, West Lafayette, Indiana 47907 USA; 2 School of Electrical and Computer Engineering, Purdue University, West Lafayette, Indiana 47907 USA; 3 Department of Physics and Astronomy, Rutgers University, Piscataway, New Jersey 08854 USA; 4 Department of Physics, Massachusetts Institute of Technology, Cambridge, Massachusetts 02139 USA; 5 National Synchrotron Light Source II, Brookhaven National Laboratory, Upton, New York 11973 USA; 6 X-ray Science Division, Advanced Photon Source, Argonne National Laboratory, Argonne, Illinois 60439 USA; 7 Center for Nanoscale Materials, Argonne National Laboratory, Argonne, Illinois 60439 USA

## Abstract

A central characteristic of living beings is the ability to learn from and respond to their environment leading to habit formation and decision making. This behavior, known as habituation, is universal among all forms of life with a central nervous system, and is also observed in single-cell organisms that do not possess a brain. Here, we report the discovery of habituation-based plasticity utilizing a perovskite quantum system by dynamical modulation of electron localization. Microscopic mechanisms and pathways that enable this organismic collective charge-lattice interaction are elucidated by first-principles theory, synchrotron investigations, ab initio molecular dynamics simulations, and in situ environmental breathing studies. We implement a learning algorithm inspired by the conductance relaxation behavior of perovskites that naturally incorporates habituation, and demonstrate learning to forget: a key feature of animal and human brains. Incorporating this elementary skill in learning boosts the capability of neural computing in a sequential, dynamic environment.

## Abstract (web summary)

Habituation is a learning mechanism that enables control over forgetting and learning. Zuo, Panda et al., demonstrate adaptive synaptic plasticity in SmNiO_3_ perovskites to address catastrophic forgetting in a dynamic learning environment via hydrogen-induced electron localization.

## Introduction

**Fig. 1:**
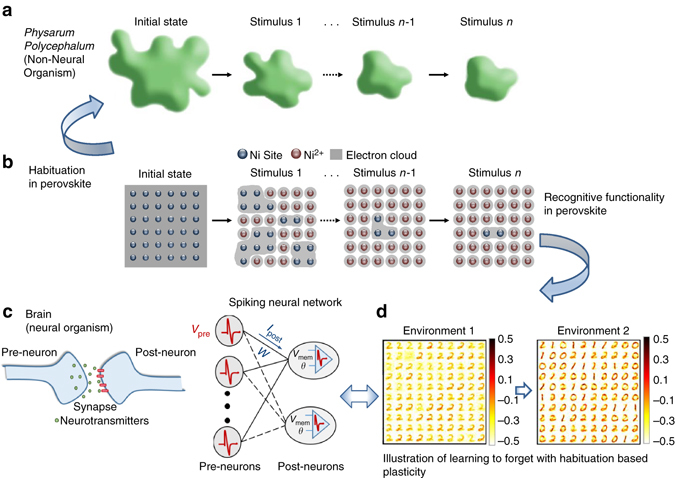
Quantum material showing habituation behavior observed in neural and non-neural organisms. **a** Nonassociative habituation learning observed in *Physarum polycephalum*. When exposed to stimulus, a diminished response is observed indicative of habituation. **b** Schematic showing the habituation process in a perovskite SmNiO_3_ (*SNO*). Between repeated stimuli (H_2_), the dynamics of carrier localization subsides, showing both non-neural habituation and neural synaptic plasticity. **c** Associative spike-timing based learning observed in a biological neural system (brain) responsible for memory formation. In the brain, synaptic plasticity is modulated by chemical transmitters, and is a function of the relative timing difference between the post and pre-neuronal spikes. The biological neural system is implemented as a Spiking Neural Network (*SNN*) that consists of a fully connected array of pre-neurons and post-neurons. The pre-neuronal voltage spike (*V*_pre_) is modulated by the synaptic weight (*w*) to generate the resulting post-synaptic current (*I*_post_). The post-neuron integrates the current that results in an increase in its membrane potential (*V*_mem_) and spikes when the potential exceeds a certain threshold (*θ*). **d** In environment 1, the SNN was presented with different images of digit 2 and learnt several patterns corresponding to the given image. In environment 2, the SNN was presented with images of digits 0 and 1. Incorporating habituation-based nonassociative learning with standard associative spike-timing dependent plasticity (*STDP*) enables the SNN to learn new patterns without catastrophic forgetting in a resource-constrained dynamic input environment. The color intensity of the patterns are representative of the value of synaptic weights with lowest intensity (*white*) corresponding to a weight value of −0.5 and highest intensity (*black*) corresponding to 0.5

Habituation, one of the primary universal learning mechanisms, can be simply defined as the decrement in response to repeated stimuli. Habituation is seen as the simplest learning form exhibited by organisms, like sea slugs^1^ and fruit flies^2^, to more complex living forms, such as rats and humans^3, 4^, and is fundamental to how an organism responds and adapts to its environment thereby increasing its chances of survival. Habituation can help animals, for instance, to focus on important stimuli for novelty detection and thus can be viewed as an integral part of attention and learning^5, 6^, and has recently been demonstrated in the single-celled non-neural organism *Physarum polycephalum*, commonly known as the slime mold^7^. In non-neural organisms, habituation is manifested by a change in global shape of the system (Fig. 1a). In more complex organisms that possess a nervous system, habituation has been shown to result from the decreased release of chemical transmitters at synaptic terminals^1, 8^. This changes the weights of certain neural connections, a mechanism known as synaptic plasticity.

The perovskite oxide compound SmNiO_3_ (SNO) is a framework of tilted NiO_6_ octahedra where Sm^3+^ ions occupy 12-fold oxygen coordinated sites and balance the charge^9^. Hydrogen doping from the environment into lattices using catalytic electrodes occurs in a reversible manner leading to massive nonlinear changes in electronic properties^10, 11^. Accompanied by incorporation of a proton, an electron can be injected into an unoccupied Ni *e*_g_ orbital. Strong Coulomb interaction existing in *e*_g_ orbitals generates a large transport gap via strong correlation effects^12, 13^. As shown in Fig. 1b, after first exposure to H_2_ (environmental stimulus 1), a significant fraction of Ni is reduced to Ni^2+^, manifested by electron localization. On changing the environment (in this case by air exposure) for a short period of time followed by re-exposure to H_2_, additional protons are incorporated into SNO, but with slower kinetics, and keep diminishing. While the perovskite mimics habituation, the varying conductance due to the correlated interactions shows inherent plasticity that can emulate biological synapses of neural organisms that are capable of more complex functionalities. Based on this discovery, we design a learning mechanism we term adaptive synaptic plasticity (ASP) that augments traditional neural systems with the key ability of learning to forget for robust and continuous learning in a dynamically evolving environment (Supplementary Fig. 1).

Figure 1c shows the underlying plasticity mechanism for memory formation and learning in the brain^14^, commonly modeled with Spiking Neural Networks (SNNs)^15^. SNNs are equipped with self-learning mechanisms such as spike-timing dependent plasticity (STDP) for real-time interaction with the environment^16–18^. However, in its naive form STDP implies that any pre/post spike pair can modify the synapse, potentially erasing past memories abruptly, commonly referred to as catastrophic forgetting^19^. This phenomenon often results in severe loss of previous knowledge in a neuromorphic system that is continuously exposed to new information (see Discussion section and Supplementary Note 1). Recent work suggests that the brain actively erases memories while learning to continuously process new environmental stimuli^20^. Due to limited storage space available, the brain forgets already learnt connections gradually to associate them with new data.

Here, we present the discovery of environmental habituation-based plasticity in SNO, which is rigorously explained by a comprehensive discussion of X-ray scattering, first-principles calculations, and ab initio dynamical simulations. Our ASP, inspired by the perovskite organismoid’s variable conductance, offers a solution to the problem of catastrophic forgetting. Incorporating habituation, a nonassociative process of adaptation seen in living organisms, into ASP learning facilitates the gradual degradation or forgetting of already learnt weights to realize new and recent information while preserving some memory about old significant data. Figure 1d shows learning to forget with ASP-based weight modulation by maintaining a balance between forgetting and immediate learning to construct a stable plastic^21^ self-adaptive SNN for dynamic environments.

## Results

### Demonstration of habituation in nickelate thin film devices

**Fig. 2:**
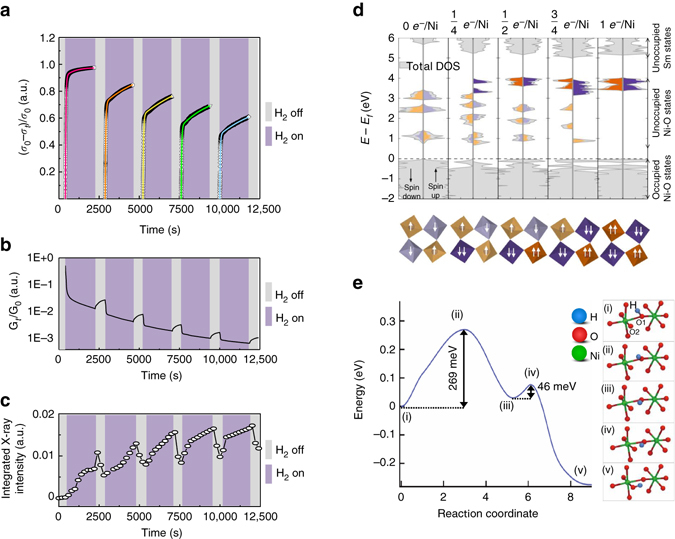
Mechanism of habituation in a perovskite nickelate. **a** In situ visualization of habituation phenomenon, i.e., exponential decrease of conductivity change upon environmental exposure (the *dots* represent the experimental data and the *solid lines* are fits.). *σ*_0_ and *σ*_*t*_ are initial and dynamical conductivity, respectively. **b** The conductance changes in response to different environments (decrease in H_2_ and increase in air) showing inherent plasticity similar to what is observed in biological synapses. *G*_0_ and *G*_t_ represent initial and dynamical conductance, respectively. **c** Structural lattice breathing monitored by in situ synchrotron X-ray diffraction. The integrated intensities of x-ray diffraction peak at *q*_z_ = 2.98 Å^−1^ related to H-SmNiO_3_ (*H-SNO*) are shown (see Supplementary Fig. 4). **d** First-principles calculation of electron-doped SNO. The *upper* figure shows density of states (*DOS*), in *gray*, at different doping levels from 0-1 added *e* ^−^ per Ni site. The unoccupied projected DOS (*PDOS*) on each nickel site is shown in *orange* and *purple*. The difference in the total DOS and the PDOS is due to the strong hybridization of the Ni and O states resulting from the covalent nature of the NiO_6_ octahedra. The *lower* figure shows the occupied Ni *e*_g_ levels for the corresponding doping levels. Same color legend is used and the *darker colors* indicate Ni with two occupied *e*_g_ states. **e** Atomic-scale pathway, and the associated energy barriers for proton migration between two neighboring O atoms labeled as O1 and O2 in (i) within a NiO_6_ octahedron in a monoclinic SNO crystal. The potential energy along the most preferred diffusion pathway (as obtained from nudged-elastic band density functional theory (*DFT*) calculation) is shown on the *left*, while selected configurations along this pathway labeled (i)–(v) are depicted on the *right*

The initial state of the system is perturbed by exposure to a new environment (namely H_2_). Electron doping via splitting of H_2_ into protons and electrons results in the reduction of several Ni sites to Ni^2+^, which is verified by X-ray spectroscopy, causing a large decrease in conductance, which can be reversed due to the weak binding of the dopant with the lattice. The temporal conductivity relaxation stems from the dynamics of surface exchange and diffusion of protons, and can be modeled as an exponential relaxation that is common to thin film devices^22^. Partial reversal of doping by withholding the H_2_ exposure for a short period of time followed by re-exposure to H_2_ and so forth leads to habituation manifested by a gradual reduction in response (Fig. 2a, Supplementary Figs. 2 and 3). Figure 2b shows the exponential change in conductance of the perovskite in different environments that motivates ASP learning. While the electron localization is the origin of the conductance change, the lattice breathes hydrogen as seen in the in situ synchrotron studies on identical devices (Fig. 2c).

### Theoretical calculations of electronic structure change

To provide a microscopic understanding of doping-driven electronic structure modification, we have carried out first-principles calculations on SNO (see Supplementary Figs. 5 and 6, and Supplementary Table 1 in Supplementary Note 2 for results on various magnetic orderings), as shown in Fig. 2d, primarily focusing on the addition of charge and the subsequent opening of the gap. We considered the doping of a pristine SNO state with all Ni^3+^, based on observations of Pbnm symmetry (where all Ni sites are equivalent) at room temperature^23^. With addition of electrons one-by-one to the $\sqrt 2$ × $\sqrt 2$ × 2 supercell of SNO, we investigated the changes in the structure and electronic energy levels. Each added electron localizes on a Ni^3+^ ion and the surrounding oxygen octahedron, shifting the lowest unoccupied orbital over 3 eV by onsite correlation to form high-spin Ni^2+^ (see Supplementary Table 2), which is consistent with variable-angle ellipsometry measurements^10^. This charge transfer into the Ni *e*_g_ orbitals is expected from the observed changes in electron filling manifested as a shift in spectral weight in the X-ray absorption data (Supplementary Fig. 7b); however, a detailed comparison requires including strong core-hole effects, which is beyond the scope of density functional theory (DFT). Thus, the fully doped case (1*e* ^−^/Ni) shows a significantly increased band gap on the order of 3 eV. The resonant magnetic coherent soft X-ray scattering measurements (RMXS, Supplementary Figs. 7c and 8) further reflect the breakdown of long-range spin order in SNO after electron doping.

### Molecular dynamics simulation of proton migration

We used ab initio molecular dynamics (AIMD) to study the mechanism of proton migration. We found that even at room temperature, the dopant hops from an oxygen atom to a neighboring one within NiO_6_ octahedron (Fig. 2e). The proton is initially bound to atom O1 (Fig. 2e, i) at a distance of approximately 2.83 Å away from the O2 atom. The proton first rotates about the O1 atom (Fig. 2e, i–iii), while being bound to O1 until the O2-H distance is lowered to approximately 1.72 Å (Fig. 2e, iii). This rotation process is associated with an energy barrier of 0.27 eV, which is lower than the typical activation barriers for H^+^ migration encountered in proton conducting perovskites (e.g., in the canonical Y-doped BaZrO_3_, ∆*E* ~0.46 eV^24^). Once the proton comes into the vicinity of O2 atom (Fig. 2e, iii), it hops over, and binds to O2 atom with a negligible energy penalty of 0.046 eV (Fig. 2e, iii–v). The proton migration between neighboring O atoms is visualized in a video in Supplementary Movie 1.

### Learning to forget with ASP

The conductance relaxation observed from Fig. 2b due to collective effects allows us to use the organismoid’s behavior to modulate synaptic plasticity for memorization and forgetting. ASP blends nonassociative habituation behavior with time-based correlation learning that helps in retention and gradual adaptation to new inputs, as well as, evokes competition across neurons to learn distinct patterns. We seamlessly integrate weight decay with traditional synaptic plasticity and modulate the leak rate using the temporal dynamics of pre- and post-synaptic neurons to realize habituation. While the temporal correlation helps in learning new input patterns, the retention of old data and gradual forgetting is attained with habituation. The ASP model for weight modulation with different windows for potentiation and depression based on the firing events of the post-/pre-neurons is shown in Supplementary Figs. 9 and 10 (see Supplementary Note 3 for details on implementation).

**Fig. 3:**
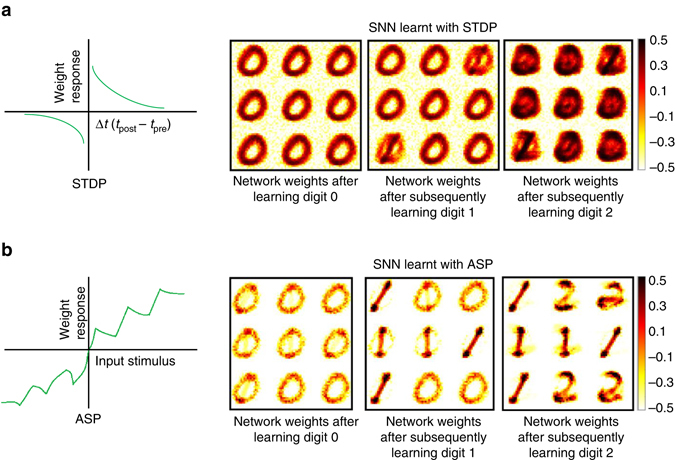
Learning by forgetting. **a**, **b** Digit representations learnt with digits 0 through 2 shown sequentially to an Spiking Neural Network (*SNN*) (with nine excitatory neurons) trained with standard spike-timing dependent plasticity (*STDP*) (**a**) and adaptive synaptic plasticity (*ASP*) that integrates habituation (**b**). Presenting the digits one-by-one sequentially i.e., first all the images for digit 0 followed digit 1, and so on can be treated as a dynamic learning environment. No particular digit instance or class is re-shown to the network. SNN trained with STDP tried to learn the new digit representation (for instance, digit 1) while retaining a portion of the old data (for instance, digit 0). However, fixed network size and absence of data reinforcement (i.e., no old data or digit showing with the new data) resulted in accumulation causing new weight updates to coalesce with already learnt patterns rendering the network incapable of categorizing the digits. In sharp contrast, ASP-learnt SNN, with identical resource constraints in place, gracefully forgets old patterns and adapts to learn new inputs effectively without catastrophically erasing old data. Supplementary Fig. 10 shows the representations learnt for a larger network when all digits 0 through 9 are presented. The color intensity of the patterns are representative of the value of synaptic weights with lowest intensity (*white*) corresponding to a weight value of −0.5 and highest intensity (*black*) corresponding to 0.5

To demonstrate the effectiveness of the organismoid-inspired learning paradigm, against standard STDP, a fixed-size SNN (with nine excitatory neurons) was trained in a dynamic digit-recognition environment, wherein digits 0 through 2 were presented sequentially with no digit re-shown to the network. Figure 3a, b shows the representations learnt with traditional exponential STDP learning^25^ against the adaptive plasticity-based learning. We see that as the network is shown digit 1, ASP-learnt SNN forgets the already learnt connections for 0 and learns the new input. Learning is more stable as neuronal connections corresponding to the older pattern 0 are retained while learning 1. ASP adopts a significant- and latest data driven forgetting mechanism (incorporated via the leak/decay phase shown in Supplementary Fig. 10), wherein older digits are forgotten to learn new digits. Hence, when the last digit 2 is presented to the ASP-learnt SNN, the connections to the excitatory neurons that have learnt digit 0 are forgotten in order to learn the latest digit 2 while the connections (or neurons) corresponding to recently learnt digit 1 remain intact.

## Discussion

Habituation is referred to as diminishing of response to a frequently repeated stimulus in the organismic biology literature^26^. We explain the principles further in this section to enable a general platform for materials design, and illustrate the experimental envelope and dynamics. To achieve plasticity in the perovskite, we vary the environment dynamically without allowing the system to achieve equilibrium. For instance, if we simply expose the perovskite at 50°C to hydrogen indefinitely, the resistance will increase to a self-limiting value and saturate over a period of several hours. Instead, we perturb the system well before saturation and then re-expose to the environment before complete reversal to the original state. In this manner, the environment is dynamically modulated over appropriate time scales quite similar to studies conducted on organisms. The perovskite retains memory of the previous exposure since not all of the dopant has left the lattice and is therefore continuously modified, leading to diminished response. The perovskite nickelates show large nonlinear changes in conductance upon electron doping due to strong correlations in the partially filled *e*_g_ Ni orbitals, enabling the mimicry of environmental plasticity. Other materials that show nonlinear changes of functional properties in response to external stimuli may be considered to further investigate similar organismic behavior. Similarly, for reversibility, weak binding of the dopant to the lattice and escape back into the environment is important. The time scale in our experiments is chosen, in part, to be in the range of experimental studies in biology, and is also close to what is noted for decision making in ant colonies, bees, and related species where environmental chemical traces (i.e., diffusion of gases or scents over sensory distances^27, 28^) are used for foraging food or collective locomotion to illustrate proof-of-principle. As a comparison, the response of neural connections in the brain is of the order of milliseconds and electronic memory is of the order of sub-microseconds. To mimic such faster time scales, instead of varying the gas-phase species in an environmental chamber (like we have done at a synchrotron beamline), one can use thin film solid or liquid electrolytes interfaced with the perovskite or other materials systems and rapidly move protons, oxygen, lithium, or other ions into and out of the habituating material. Since ions are charged and electrons can be reversibly anchored to the partially filled d-orbitals, electric fields can be used to operate these devices that can be integrated onto circuits^29–31^. The varying conductance of the perovskite organismoid indicates an inherent plasticity that can be used for creating artificial neural systems. This motivated us to design ASP that incorporates habituation with traditional spike-timing correlation-based learning. It is implemented by modulating the exponential leak rate of the weights based on the significance of the incoming inputs, and is critical for learning to forget in a dynamic environment. As explained in Supplementary Note 4, this model is also compatible with other classes of oxide-ionic devices that incorporate filamentary switching or spin-based devices.

The ability to learn tasks in a sequential fashion is crucial to the development of neural systems. In sequential learning, during the training process no information is re-presented to the network. Such continual/sequential learning poses particular challenges for neural networks due to the tendency for knowledge of previously learnt task(s) (e.g., task A) to be abruptly lost as information relevant to the current task (e.g., task B) is incorporated. This phenomenon, termed catastrophic forgetting^19, 32, 33^, occurs specifically when the network is trained sequentially on multiple tasks because the weights in the network that are important for task A are changed to meet the objectives of task B.

Typically, SNNs use STDP to modify the synaptic weights for unsupervised learning of inputs. However, memory persistence is a prominent problem that has been well-documented with STDP as it implies that any pre/post spike pair can modify the synapse, potentially erasing past memories abruptly leading to catastrophic forgetting^19, 32, 33^. Here, we note specifically that catastrophic forgetting will occur only when considering sequential learning of tasks.

In our experiments with MNIST digit recognition, we show each class/digit sequentially thereby creating a dynamic learning environment (refer to Methods for details). Presenting the digits one-by-one sequentially, i.e., first all the images for digit 0 followed by digit 1, and so on, can be treated as a dynamic learning environment. No particular digit instance or class is re-shown to the network. Thus, even with proper STDP tuning and slower learning rate, we cannot still unlearn the already learnt digits, when new patterns are shown to the network. This results in overlap of representations as seen in Fig. 3 and Supplementary Fig. 10. The decay mechanism incorporated with ASP (utilizing habituation) in turn helps in unlearning or forgetting the previously learnt data to learn new patterns without any overlap.

Furthermore, in order to prevent catastrophic forgetting in STDP-learnt SNNs, the network is generally re-trained with both the new and the old information (already learnt) when the network has to learn a new class. Old information re-presented with new data during training ensures that the latter or new input data do not replace previous patterns. However, in online real-time learning, it is often impractical and even expensive to store all old data samples for re-training, each time a new input pattern is encountered. ASP owing to its forgetting while learning capability offers a promising solution for real-time dynamic learning without the expensive re-training procedure.

## Methods

### Growth of epitaxial perovskite oxide thin films

LaAlO_3_ (001) single crystals were used as substrate for epitaxial growth of SNO thin film by magnetron co-sputtering of metallic nickel in direct-current mode (at power of 75 W) and samarium targets under radio-frequency mode (at power of 150 W) in 5 mTorr of argon and oxygen mixture flowing at rate of 40/10 standard cubic centimeters per minute (sccm). The as-sputtered sample was then transferred into a custom-built high-pressure vessel and annealed under 100 bar of pure oxygen at 500 °C for 24 h in a tube furnace.

### Electrical characterization

In situ temporal resistance measurements were done in a sealed custom-designed chamber equipped with a gas flow controlling system, from which we could switch the chamber inner atmosphere between various environments. In this experiment, we use 5% H_2_ balanced by 95% argon as the stimulus environment, and use air to remove the hydrogen stimulus. The habituation only requires one stimulus (hydrogen), similar to what is seen in real organisms. Four 100 nm Pt strips (0.5 mm × 5 mm) were deposited on the top of epitaxial SNO thin films by electron beam evaporation. The strips function as catalyst to split H_2_ into protons and electrons for the electron doping of SNO, and electrical contacts for the resistance measurements as well. The distance between each Pt strip is 1 mm. The experiments were conducted at 50 °C to maintain a steady temperature throughout the studies. Resistance was calculated from current-voltage curves swept between −0.1 to 0.1 V using Keithley 2635A instrument. The initial chamber atmosphere was air, and Pt/SNO sample was placed onto the sample stage for 30 min to reach stable temperature. Real-time resistance testing, was conducted using a custom LabVIEW code. To avoid temperature fluctuation during gas switching, the 5% H_2_/95% Ar was made to flow to the chamber at a moderate flow rate of 30 sccm. After a 30 min reaction, the gas was switched back to air by making dry air flow at the same flow rate (30 sccm). After 10 min, gas was changed to 5% H_2_/95% Ar again, and this H_2_-Air cycle was repeated. The relaxation of conductivity after exposure to environment is fitted with the formalism to study conductivity relaxation in thin film devices^34, 35^, (*σ*_0_ − *σ*_t_)/*σ*_0_=*C* − *A*_1_exp(−*k*_1_ *t*) − *A*_2_exp(−*k*_2_ *t*), where *σ*_0_, and *σ*_t_ represent initial and dynamical conductivity, respectively, *k*_1_ and *k*_2_ depicting the relaxation kinetics.

### In situ synchrotron X-ray diffraction

Synchrotron X-ray diffraction measurements of the SNO devices were conducted at an insertion device beamline, 12ID-D at the Advanced Photon Source, Argonne National Laboratory, on a six-circle Huber goniometer with an X-ray energy of 20 keV using a pixel array area detector (Dectris Pilatus 100 K). The X-ray beam had a flux of 10^12^ photons per second. The *q*_z_-scan (*L*-scan) was obtained by removing the background scattering contributions using the two-dimensional images. For in situ X-ray diffraction testing, Pt/SNO sample was placed in a testing compartment sealed by Kapton tape. The testing condition followed the atmosphere progression mode shown in Fig. 2a.

### Resonant magnetic coherent soft X-ray scattering

RMXS study was performed at the beamline 23-ID-1 of the National Synchrotron Light Source II (NSLS-II), at Brookhaven National Laboratory. All data were collected using horizontally polarized light and a vertical scattering geometry, with photon energy tuned near the Ni-*L*_3_ absorption edge. The probing geometry is illustrated in Supplementary Fig. 7a. The pristine SNO thin film is patterned with Pt stripes and hydrogen is intercalated to yield electron-doped regions H-SmNiO_3_ (H-SNO) of width 0.1 mm. The magnetic scattering signal is measured by a two-dimensional charge-coupled device (CCD) positioned 33 cm from the sample, while the X-ray absorption is collected in total fluorescence yield, also using the CCD (away from structural or magnetic reflections). In order to reach the magnetic reflection, at **Q** = (1/4,1/4,1/4), the sample was oriented so that the scattering plane is spanned by crystal vector [111] and [1-10]. The film was illuminated by a coherent X-ray beam whose coherent fraction is selected by a 10 μm diameter pinhole in close proximity to the sample. The measurements were performed at ~20 K which is well below the Neel temperature of SNO (~200 K).

### First-principles calculations for SNO electronic structure

First-principle calculations were carried out within the DFT+U approximation with the Vienna ab initio Simulation Package (VASP) code^36, 37^ using the projector augmented plane-wave (PAW) method of DFT^38^ and the supplied pseudopotentials: Sm_3 (valence: *5s* ^2^ *5p* ^2^ *6s* ^2^ *4f* ^1^), Ni_pv (valence: *3p* ^6^ *4s* ^2^ *3d* ^8^) and O (valence *2s* ^2^ *2p* ^4^ *)*. To treat the exchange and correlation, the PBE functional was used within the generalized gradient approximation (GGA)^39, 40^ and the rotationally invariant form of DFT+U of Liechtenstein et al.^41^ with *U* = 4.6 eV and *J* = 0.6 eV. For structural determination of pristine SNO, we started with the Materials Project structure^42^ added a small monoclinic distortion (*β* ≈ 90.75°) and allowed the cell and ionic positions to relax until the forces were less than 0.005 eV/Å on each ion. All calculations were carried out with the tetrahedral method with Blöchl corrections^43^, a 6 × 6 × 4 Monkhorst-Pack *k*-point mesh for the $\sqrt 2$ × $\sqrt 2$ × 2 supercell, and a plane-wave energy cutoff of 500 eV. When simulating electron doping, extra electrons were added to the calculation with a positive background compensation charge. For SNO, we added 1, 2, 3, or 4 electrons to the monoclinic $\sqrt 2$ × $\sqrt 2$ × 2 supercell with a G-type magnetic ordering, resulting in an electron-doping concentration of 1/4, 1/2, 3/4, 1 *e* ^−^/Ni, respectively. In each case, we allowed the internal ionic positions to relax, using the same force tolerance as before, while keeping the volume and cell shape unchanged. When studying effect of magnetic order on the fully doped case (Supplementary Fig. 5), both the ionic positions and the lattice parameters were relaxed.

### Ab initio molecular dynamics simulations

We performed AIMD simulations within GGA with Hubbard correction using the PAW formalism as implemented in VASP^36, 37^. The computational supercell consists of four unit cells of monoclinic SNO (2 × 2 × 1 repetitions of unit cell; 80 atoms). Periodic boundary conditions are employed along all directions. The exchange correlation is described by the PBE functional^39, 40^, with the same pseudopotentials as used in the electronic structure calculations. The plane-wave energy cutoff is set at 520 eV. The Brillouin zone is sampled at the *Γ*-point only. Using AIMD simulations in the isobaric-isothermal (NPT) ensemble, we first thermalize the SNO computational supercell at various temperatures ranging from 300–800 K and zero external pressure for 10 ps using a timestep of 0.5 fs. During these simulations, the cell volume, cell shape, as well as the atomic positions are allowed to vary via the Parrinello-Rahman scheme^41^; the temperature conditions are maintained by using a Langevin thermostat^44^. Next, we insert a proton within the thermalized SNO (at a given temperature), such that it is at a distance of 0.98 Å away from an arbitrarily chosen O atom. Note that we ensure supercell neutrality upon addition of the proton via a background negative charge. To monitor the diffusion of the inserted proton (Supplementary Movie 1, Supplementary Note 5), we perform AIMD simulations at constant volume (and shape) and temperature (i.e., NVT ensemble) for an additional 10 ps. For these AIMD simulations of doped SNO, constant temperature conditions are maintained via Nose Hoover thermostat^44^ as implemented in VASP.

### Simulation methodology for SNNs

The ASP learning algorithm was implemented in BRIAN^45^ that is an open source large-scale SNN simulator with parameterized functional models (Leaky-Integrate-and-Fire) for spiking neurons. We used the hierarchical SNN framework (Supplementary Fig. 9) to perform digit recognition with the MNIST dataset^46^. The network topology and the associated synaptic connectivity configuration were programmed in the simulator. The spiking activity (or time instants of spikes) of pre- and post-neurons were monitored to track the corresponding pre-/post-synaptic traces that were used to estimate the weight updates in the recovery/decay learning phase of ASP.

### Data availability

The data that support the findings of this study are available from the corresponding author upon reasonable request.

## Electronic supplementary material

Supplementary Information Supplementary Figures, Supplementary Tables, Supplementary Notes and Supplementary References

Supplementary Movie 1 Ab initio molecular dynamics simulations showing migration of proton in H‐doped monoclinic SNO crystal at 300 K. The proton hops from one O atom to another neighboring O atom within the NiO6 octahedron in a facile manner (see Fig. 2e for details on the activation barriers). The Ni, O, Sm and H are depicted as green, red, yellow, and blue spheres respectively. For the sake of clarity, only the hydrogen, and the Ni/O atoms belonging to the two NiO6 octahedra closest to the hydrogen are shown as large spheres; the atoms far away from the hopping phenomena are depicted with small translucent spheres. Our AIMD simulations at 300 K at various H doping levels show that the SNO lattice monotonically expands with addition of hydrogen approaching lattice expansion of ~5% for 1 H per unit cell of SNO
