# Supplementary material for: Habituation based synaptic plasticity and organismic learning in a quantum perovskite
Source: Nat Commun. 2017 Aug 14;8:240. doi: 10.1038/s41467-017-00248-6 (PMC5556077; doi:10.1038/s41467-017-00248-6)
Supplement: Supplementary file 1 — Supplementary Figures, Supplementary Tables, Supplementary Notes and Supplementary References [file 41467_2017_248_MOESM1_ESM.pdf]

File Name: Supplementary Information
Description: Supplementary Figures, Supplementary Tables, Supplementary Notes and Supplementary References

File Name: Supplementary Movie 1
Description: Ab initio molecular dynamics simulations showing migration of proton in H-doped monoclinic SNO crystal at 300 K. The proton hops from one O atom to another neighboring O atom within the NiO_6 octahedron in a facile manner (see Fig. 2e for details on the activation barriers). The Ni, O, Sm and H are depicted as green, red, yellow, and blue spheres respectively. For the sake of clarity, only the hydrogen, and the Ni/O atoms belonging to the two NiO_6 octahedra closest to the hydrogen are shown as large spheres; the atoms far away from the hopping phenomena are depicted with small translucent spheres. Our AIMD simulations at 300 K at various H doping levels show that the SNO lattice monotonically expands with addition of hydrogen approaching lattice expansion of ~5% for 1 H per unit cell of SNO.

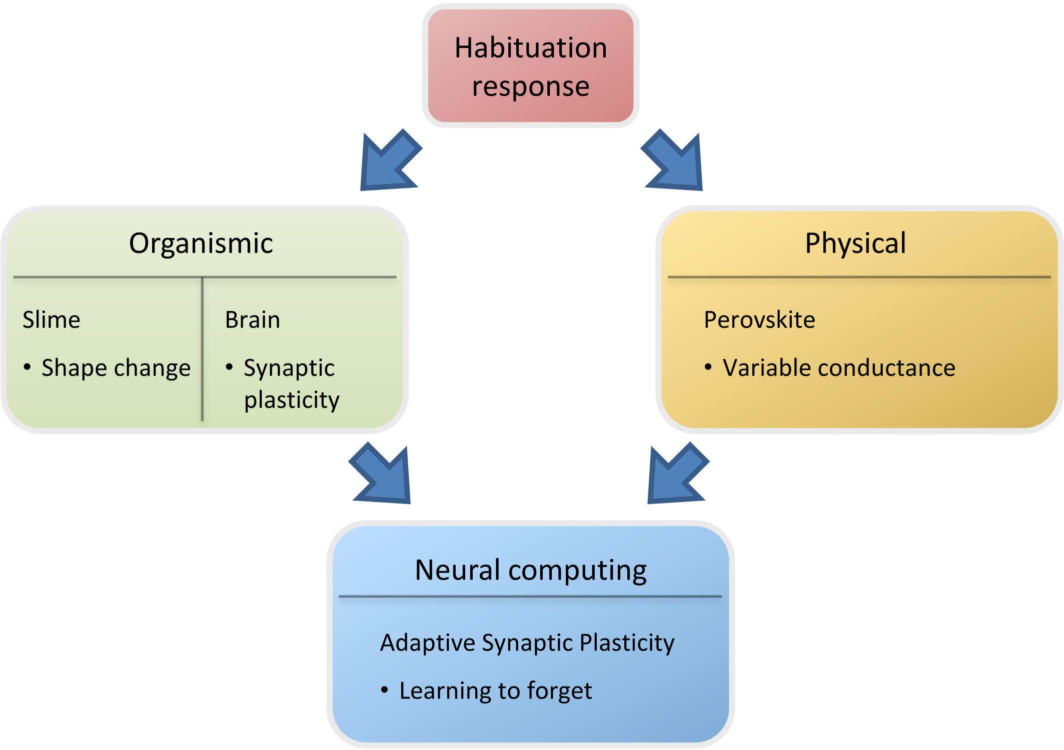

Supplementary Figure 1 | Habituation response in the perovskite and learning mechanism in neural/non-neural organisms. Habituation is a ubiquitous behavior present across the phyla of living beings that help organisms to learn and adapt to different aspects of the environment. It has been demonstrated to cause short-term and long-term potentiation of synaptic connections (or synaptic plasticity) that is key to memory formation in neural organisms. In non-neural organisms such as slime, habituation is seen as a change in its shape in response to different environments. The perovskite's non-linear response to the environment (H_2 and Air in this study) with varying conductance mimics simple adaptation behavior and motivates the Adaptive Synaptic Plasticity (ASP) learning (see Supplementary Fig. 10). In ASP, we incorporate habituation by weight leaking coupled with traditional spike timing correlation to demonstrate learning to forget for robust and stable learning of artificial neural systems.

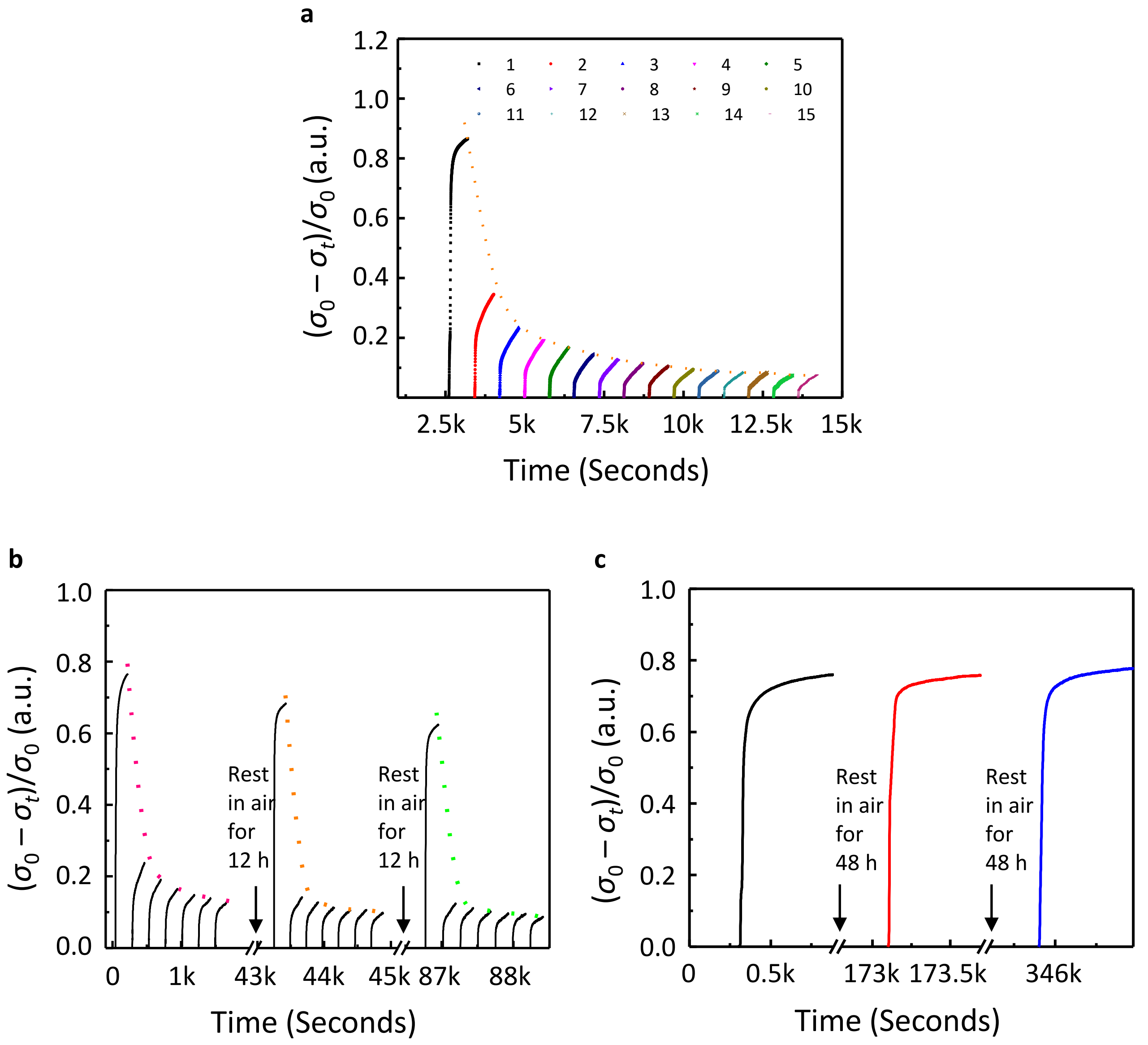

Supplementary Figure 2 | Habituation-forgetting-habituation and complete recovery behavior of $SmNiO_3$ (SNO) device. In organisms, if the stimulus is withheld for extended period of time, the original state can be recovered. Subsequent exposures to the environment will result again in habituation. Strikingly identical to this behavior, our nickelate devices can be made to forget previous exposures to hydrogen by resting in air. **a**, A set of experiments on a device after it was recovered by an air anneal then followed by testing for 15 cycles. The continuous diminished response demonstrates habituation behavior. **b**, After seven cycles of H_2/Air treatments, the SNO was left in air for 12 h. The device started to recover and approached its original state. With the same manner of H_2/Air exposure again, the habituation behavior could be reproduced. This forgetting-habit forming process was repeated by resting the SNO device in air for another 12 h and re-exposure to H_2/Air. **c**, non-habituation by fully recovering SNO. It is worth noting that if the H_2 treatment was followed by an extended exposure of the nickelate device in air for 48 h, no habituation phenomenon would be present, again similar to what is observed in experiments conducted on organisms. The dotted conducting line in (**a**) and (**b**) indicated the trend of diminished response. These experiments were conducted in a manner identical to numerous experiments conducted on organisms in the biology literature and are all cited in the manuscript and Supplemental Information files.

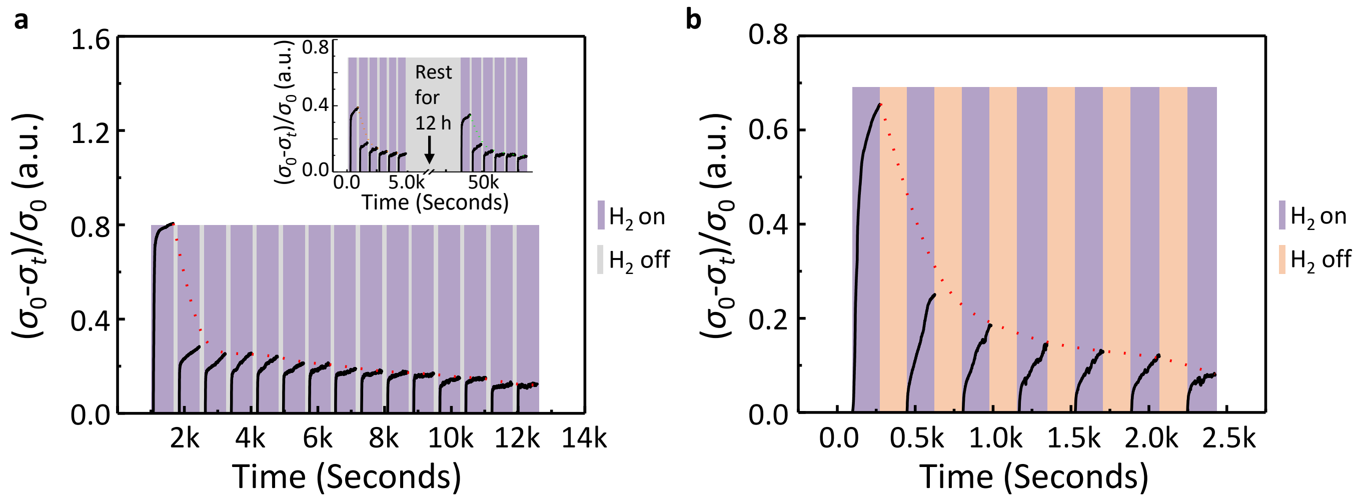

Supplementary Figure 3 | Additional habituation experiments in ambient and inert environments. **a**, A set of tests on $SmNiO_3$ (SNO) device in open ambient environment with repeatedly turning on/off H_2 for 15 cycles. The decreased response of SNO to single stimulus H_2 demonstrates the habituation behavior. The inset shows after resting in ambient environment for 12 h, the habituation behavior can be seen again. The forgetting-habituation behavior was reproduced by resting SNO for another 12 h and treating with H_2 again. **b**, Another set of experiments on SNO in a custom-designed chamber. Distinct from the experiments shown in Fig. 2a, here we have inert Argon gas background. The evident habituation behavior with hydrogen demonstrates habituation is accomplished with just one stimulus, identical to experiments conducted in real organisms. The dotted conducting line in (**a**) and (**b**) indicated the trend of diminished response.

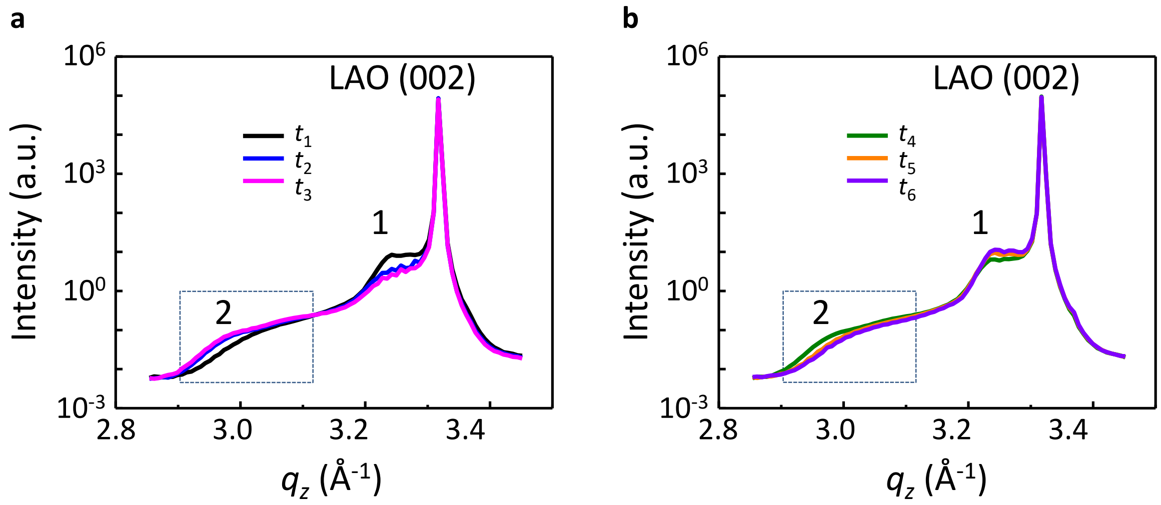

Supplementary Figure 4 | A set of representative *in situ* synchrotron X-ray diffraction patterns monitoring structural evolution upon breathing in H_2 and air sequentially. a, X-ray diffraction patterns in H_2 environment. $SmNiO_3$ (SNO) thin film and substrate $LaAlO_3$ (LAO) are indexed in pseudocubic lattice system. SNO (002) peak (labeled 1) appears close to LAO (002) peak. The peak at q_z = 2.98 Å^{-1} (peak 2) is related to $HSmNiO_3$ (H-SNO)[1]. It is clearly seen that when treated with H_2, peak 1 drops with exposure time ($t_1 \rightarrow t_2 \rightarrow t_3$), while peak 2 increases with longer exposure, indicating H-SNO phase emergence. The proton concentration can be estimated to be of the order of 0.03-0.05 doping per unit cell by comparing the intensity to that of a fully doped sample. **b**, X-ray diffraction patterns in air environment. The gas environment was switched to air and structural evolution was monitored. It is evident that no new peaks are present. An opposite trends is observed, i.e. the SNO phase is restored and H-SNO phase diminished when breathing in air. The areas of peak 2 for all measured data during H_2/air breathing are calculated via integration of the region shown in dotted line box and plotted in Fig. 2c.

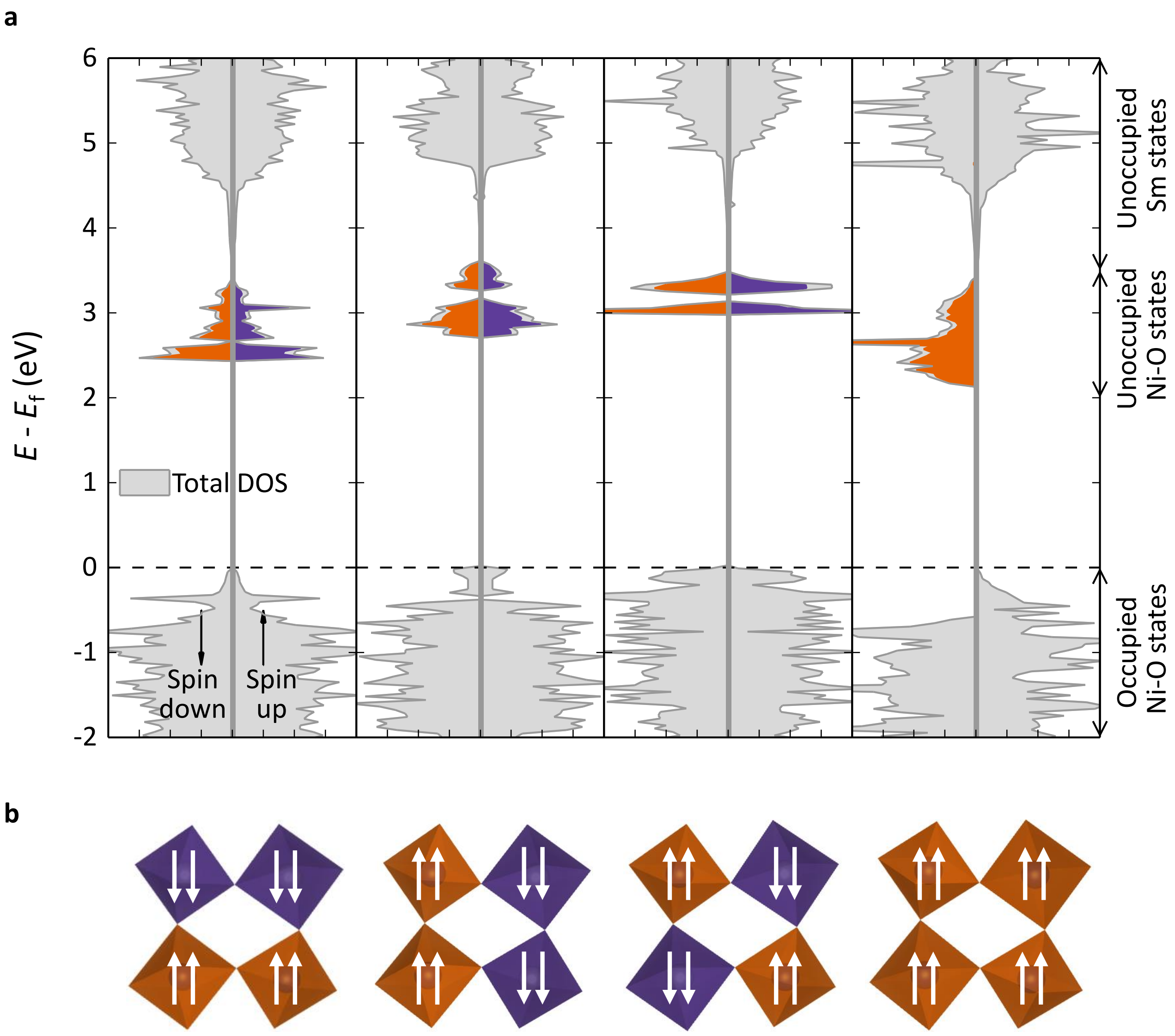

Supplementary Figure 5 | Density of States (DOS) based on different magnetic orderings of doped $SmNiO_3$ (SNO). a, DOS for fully doped ($1e^-$/Ni) SNO - all Ni^{2+} - with various magnetic orderings. Despite the magnetic ordering, all band gaps are on the same order. The structures are fully relaxed and the Ni^{2+} are in high spin configurations, which is favored over the low spin configuration due to the Hund's coupling. Colored states are the unoccupied Ni projected DOS (PDOS), corresponding to e_g states. **b**, The occupied Ni e_g electrons on each Ni site for each magnetic ordering. The colors of the octahedra correspond to the colored PDOS in (**a**).

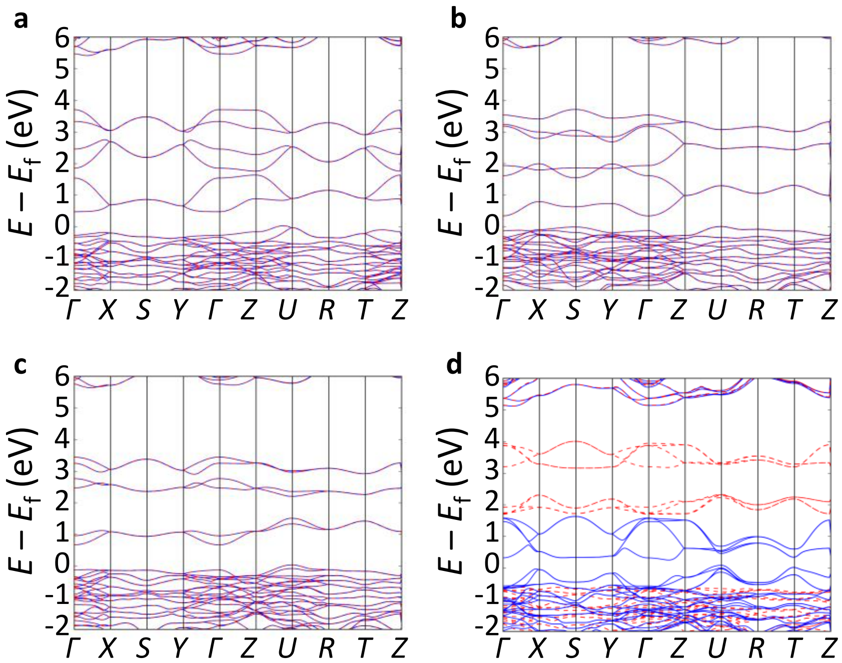

Supplementary Figure 6 | Band structure of the monoclinic structure with Jahn-Teller distortion. **a-d** show the band structure with (**a**) antiferromagnetic orderings with layers perpendicular to the *z*-axis (A-type); (**b**) with columns along the *z*-axis, forming a checkerboard in the *xy*-plane (C-type); (**c**) with a three dimensional checkerboard (G-type); and (**d**) ferromagnetic ordering (F-type). For A-, C-, and G-type, the bands are spin degenerate. The two highest valence bands are the 4 occupied Ni e_g electrons (one on each Ni site). The 6 bands between 0 and 4 eV are the unoccupied Ni e_g states. For F-type, the spin up bands are shown in solid blue, and the spin down bands are shown in dashed red. The four highest valence bands are the 4 occupied Ni e_g electrons (one on each Ni site). All of these bands are spin up (the four bands can be clearly seen at the *U* point), leaving 8 unoccupied spin down bands and 4 spin up. The bands between 0 and 4 eV are the unoccupied Ni e_g states.

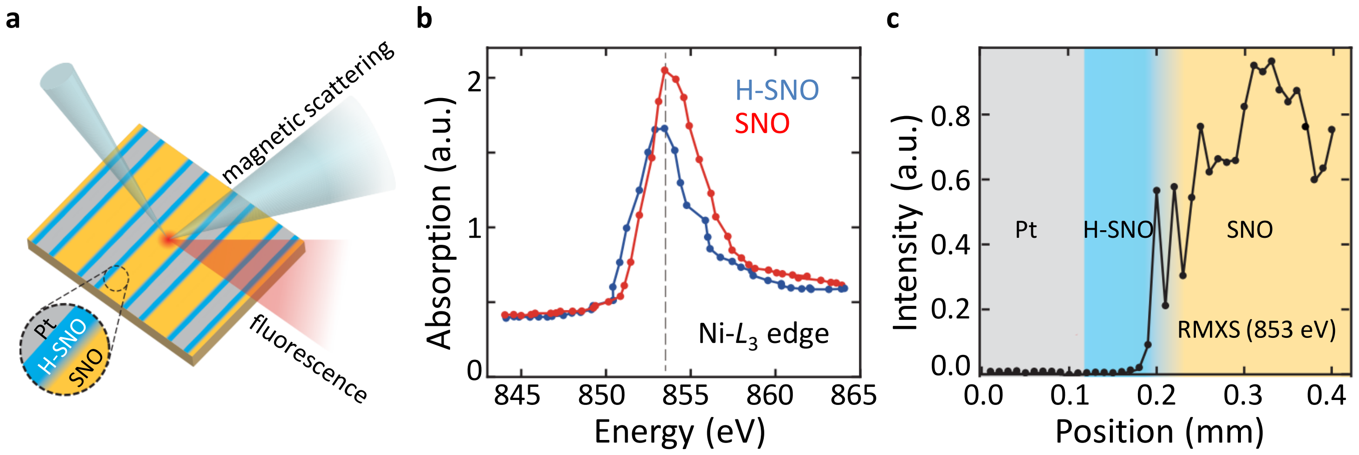

Supplementary Figure 7 | Resonant magnetic X-ray scattering (RMXS) study of $SmNiO_3$ (SNO) after breathing in H_2. a, The experimental setup at beamline 23-ID-1 (CSX-1) at the NSLS-II. In the SNO region (orange), the sample is identical to the pristine (un-doped) SNO. The film regions close to Pt bars (gray) are hydrogenated and marked as $HSmNO_3$ (H-SNO, blue). **b**, Ni-L_3 X-ray absorption profiles of H-SNO (blue) and SNO (red). The absorption peak at 853 eV in SNO corresponds to the Ni-L_3 edge as noted in literature[2]. When doped with electrons, the absorption edge shifts to lower energy, and develops a shoulder on the high energy side. This shift in spectral weight is indicative of an increase of electron filling on the Ni site suggesting doping-induced changes in the orbital filling and electronic structure. **c**, Spatial RMXS study of SNO. Position-dependent magnetic peak intensity was collected at 853 eV (dashed line in **b**). The magnetic reflection is only present in the SNO region.

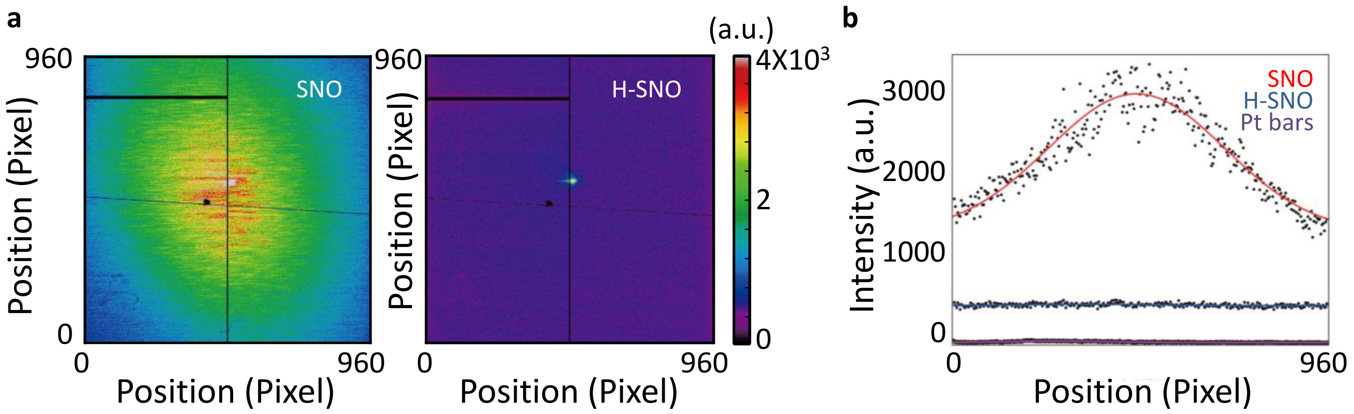

Supplementary Figure 8 | Typical magnetic scattering signal for $SmNiO_3$ (SNO) and $HSmNiO_3$ (H-SNO) region of sample after breathing in H_2. a, Detector images of the magnetic reflection peak from different regions of the sample. A diffuse magnetic peak centered at the wavevector **Q** = (1/4,1/4,1/4) can be clearly seen when the undoped SNO is illuminated (left), while no peak was found in H-SNO (right). Both measurements have been performed at 20 K, well below the Neel temperature (200 K) of $SmNiO_3$. The black line crossing the center of CCD is the shadow of wires holding beam stop (shown as a black spot near center). The color-map figure indicates the intensity of signal. **b**, Magnetic reflection signal for three different regions of the sample (SNO, H-SNO, and Pt bar), with Gaussian fit (plus baseline) overlaid. To obtain the RMXS intensity, we integrated the CCD signal along vertical slices (with 50 pixels lateral width) through the peak center in order to average out the intensity fluctuations (speckle pattern) arising from domain interference (**b**). When the beam is on the Pt bar, no signal can be measured, due to the opacity of the heavy metal layer to soft X-rays. The fluorescence background increases in the H-SNO region, where, however, no magnetic scattering can be detected. The magnetic reflection only appears in the undoped SNO region. All linecuts are fitted using a Gaussian lineshape with a uniform background. The position-dependent magnetic peak intensity can then be extracted as shown in Supplementary Fig. 7c.

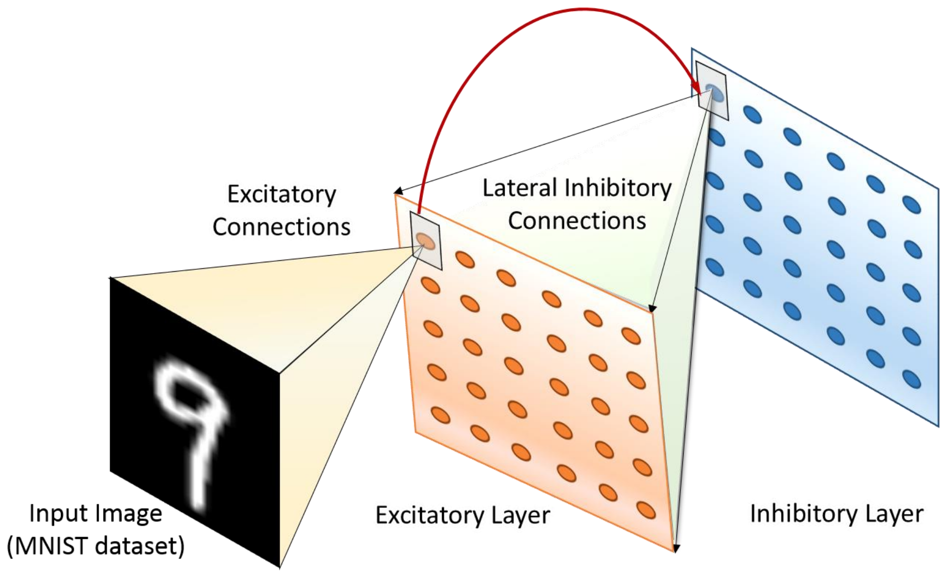

Supplementary Figure 9 | Spiking Neural Network (SNN) topology for pattern recognition consisting of input, excitatory and inhibitory layers arranged in a hierarchical fashion. The topology consists of an input layer followed by excitatory and inhibitory layers. The input layer contains 28x28 pixel image data (with one neuron per image pixel) from MNIST dataset[3]. Each input pattern or image is converted to a Poisson spike train based on the pixel intensities of the images in the dataset. The input layer is fully connected to the excitatory neurons, that are connected to the corresponding inhibitory neurons in a one-to-one manner. Each of these neurons inhibit the excitatory layer neurons except the one from which it receives the forward connection. This connectivity structure provides lateral inhibition that limits the simultaneous firing of various excitatory neurons in an unsupervised learning environment, promotes competitive learning causing them to learn different input patterns from each other. Besides lateral inhibition, we employ an adaptive membrane threshold mechanism called homeostasis[4] that regulates the firing threshold to prevent a neuron to be hyperactive. It equalizes the firing rate of all neurons preventing single neurons from dominating the response. During learning, the excitatory synaptic weights from the input layer to each excitatory neuron are modulated to learn a particular input digit using the learning rule. Towards the end of the learning phase, the weights (or excitatory connections) that are randomly initialized eventually learn to encode a generic representation of the digit patterns. Specifically, the weights fanning out of the higher-intesity (or white) pixel regions will get potentiated while the weights from the low-intensity regions on the input image will be depressed during the learning phase. Correspondingly, the color-map figures shown in Fig. 1d, Fig. 3 and Supplementary Fig. 10b represent the weight values learnt corresponding to each excitatory neuron when learning stops.

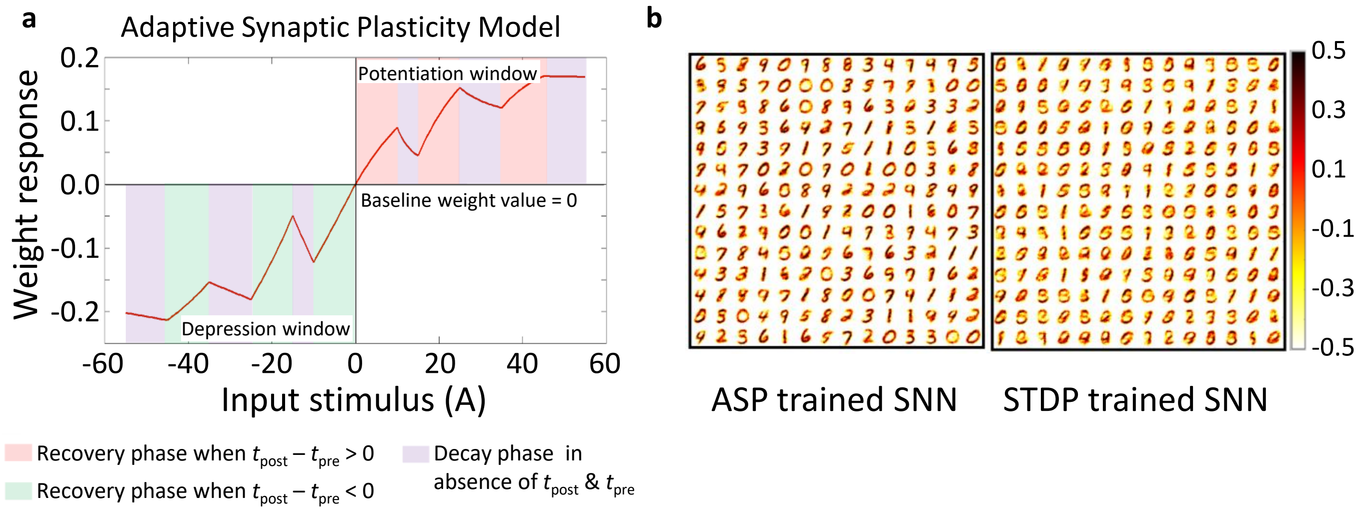

Supplementary Figure 10 | Adaptive Synaptic Plasticity (ASP) learning for weight modulation in a Spiking Neural Network (SNN). a, When a spiking activity is observed at the post/pre neuronal synaptic terminal, the recovery phase begins. This phase involves an exponential increase (potentiation) or decrease (depression) based on the temporal difference between the spiking activities of the pre- and post- neurons. The decay or forgetting phase in a synaptic weight ensues when there is no spiking activity (or input stimulus) observed at both post/pre neuron. The weights are dynamic during the training phase when input patterns are presented. The leak dynamics determine which post-neuronal connections (that have learnt old or insignificant data) should be forgotten to learn the new data. Whilst, the recovery phase potentiation/depression is geared towards making synaptic weight updates to learn a generic representation rather than learning specific training patterns. For instance, the weights of an excitatory layer post-neuron learning a digit 2 should spike for different instances of 2 so that it learns a more generic representation rather than just mimicking a specific instance. Thus, synaptic depression (based on spike timing correlation) and leak (based on habituation) have different roles in ASP learning. ASP incorporates the significance of the inputs to modulate the weights (see Supplementary Note 3 for details on implementation). That is, the weight updates during recovery phase are more prominent for frequently spiking input neuron. Also, the leak rate during the decay phase is varied taking into account the post-synaptic or excitatory neuron's spiking activity and membrane threshold $V_{\text{thresh}} + \theta$, such that recent input patterns do not overwrite old but significant data. The leak rate decreases as the weights become more and more prominent (either in potentiation or depression window), that is basically habituation. This behavior helps to retain significant information (corresponds to weights with higher negative/positive values) while forgetting (or leaking) the weights corresponding to insignificant information. **b,** To show the effectiveness of the proposed learning model for larger problems, we trained an SNN of 200 excitatory neurons with Spike Timing Dependent Plasticity (STDP) and ASP in a dynamic environment when all digits 0 through 9 are presented sequentially. To ensure that the earlier digits are not completely forgotten, the number of training instances of each digit category were arranged in a decreasing order i.e. digit 0 had more training instances than digit 1 and so on. So, the network will try to retain more significant data while learning recent patterns. It is clearly seen that the SNN learnt with our proposed ASP encodes a better representation of the input patterns in comparison to the standard STDP trained network. In fact, the network is able to represent all digits. Without habituation in STDP trained SNN, most of the representations are illegible due to substantial overlap. As noted in Supplementary Note 4, ASP learning can also be naturally integrated with filamentary switch or spin-based devices. The color intensity of the patterns are representative of the value of synaptic weights with lowest intensity (white) corresponding to a weight value of -0.5 and highest intensity (black) corresponding to 0.5.

Supplementary Note 1: Related work for habituation based learning

There have been prior efforts on integrating habituation for implementing autonomous mobile robots[5-7]. Essentially, the non-associative learning rule that represents a decreased response after exposure to repeated stimuli, has been demonstrated to be crucial for the attention phenomenon. Consequently, in Ref. 5, the authors had demonstrated that inserting such a rule at the synaptic level increased a robot's adaptation capability (that was controlled by a Spiking Neural Network (SNN)) by enabling the robot to ignore broader contextual irrelevant information. In Ref. 6, the authors investigated operant conditioning in neurobotics context in multiple learning scenarios. Specifically, in Ref. 6, the authors used the habituation enhanced synaptic plasticity to model simple operant conditioning learning in mobile robots that can perform complex behaviors with simpler neural components. On the other hand, from neuroscience perspective, habituation mechanisms (not related with synaptic plasticity) have been used to model visual cortical dynamics to better understand how the brain perceives slanted, curved surfaces and 2D as 3D objects[8,9]. The model shows how chemical transmitters that habituate in an activity dependent manner trigger attention that eventually affects 3D/2D perception. Our work, in contrast to the above studies, integrates habituation with synaptic plasticity to emulate the forgetting capability of the brain in order to build a stable-plastic self-adaptive SNN for dynamic environments without catastrophic forgetting. Adaptive Synaptic Plasticity (ASP) learning facilitates the gradual degradation or forgetting of already learnt weights to realize new and recent information while preserving some memory about old significant data. While the current work focuses on a visual recognition application, the ASP learning rule can serve as a general unsupervised learning model across neuromorphic applications that addresses the catastrophic forgetting issue.

Supplementary Note 2: First-Principles calculation of SNO band structure

The undoped (pristine) calculation are carried out on monoclinic SNO with a Jahn-Teller distortion, relaxed from a Pbnm structure and freezing in a monoclinic distortion with $\beta \neq 90$. This structure (space group $P2_1/n$) is a $\sqrt{2}$ x $\sqrt{2}$ x 2 supercell with two inequivalent Ni sites. Considering 10 atom/cell magnetic orderings for the monoclinic structure, we found the energies of all these relaxed structures to be within 0.1 eV/Ni of each other with band gaps all smaller than 1 eV (See Supplementary Table 1 for further details). Consequently, the specific choice of the underlying magnetic structure in the spin-polarized DFT+U is not expected to influence appreciably the evolution of the band structure with doping. Previous computational work on rare earth nickelates, employing both dynamical mean field theory (DMFT) and DFT+U, has shown that the insulating phase exhibits a disproportionated structure, which is often exaggerated by DFT+U[10]; furthermore, room temperature experiments show only a small[11] or no[12] disproportionation. For each added electron, its localization can be observed through the magnetic moment of the Ni, the oxygen octahedral size or the PDOS of the Ni. The localized electron on a Ni site resulting in a high-spin Ni^{2+}, where Hund's rules are favored over a Jahn-Teller distortion. This can be clearly seen for 1/4 and 1/2 e^-/Ni in Supplementary Table 2. The octahedral distortions observed for 3/4 and 1 e^-/Ni are also constrained by the volume of the cell, which is not allowed to change in the ionic relaxation in these calculations.

Supplementary Table 1:
Total energies, band gaps, lattice vectors and non-orthorhombic angle of the 20 atom/cell of the monoclinic structures. A, C, G and F are 10 atom/cell antiferromagnetic orderings of the monoclinic structure.

Magnetic Order	Total Energy (eV)	Gap(eV)	a (Å)	b (Å)	c (Å)	β (°)
A	-134.80	0.54	5.300	5.724	7.481	90.007
C	-134.64	0.43	5.270	5.812	7.441	90.011
G	-134.59	0.75	5.278	5.818	7.421	90.005
F	-135.01	0.30	5.328	5.553	7.600	90.031

Supplementary Table 2:
Magnetic moment of each of the four Ni and properties of the compassing oxygen octahedron in the doped SNO structures with G-type magnetic ordering. We elected to keep the overall volume of the calculations fixed as in reality the electron doping will not occur the same regularity present in the calculation. While the overall octahedral tilt pattern is not affected by electron doping, the tilt angles become more acute. When the lattice parameters are allowed to relax the overall volume increases; however, the octahedral volume increases that the tilt angles become even more acute.

Property	Ni_1	Ni_2	Ni_3	Ni_4
**0 added electrons per Ni**				
Magnetic Moment (μ_B)	0.92	-0.92	0.92	-0.92
Octahedral Volume (Å^3)	10.62	10.62	10.62	10.62
Ni-O Distances (Å)	2.17, 1.91, 1.93	1.91, 2.17, 1.93	1.91, 2.17, 1.93	2.17, 1.91,1.93
Ni-O-Ni_B Angle (°) (Ni_B)	148.8 (Ni_2)	148.6 (Ni_3)	148.8 (Ni_4)	148.6 (Ni_1)
**1/4 added electrons per Ni**				
Magnetic Moment (μ_B)	0.81	-0.84	0.86	-1.65
Octahedral Volume (Å^3)	10.46	10.48	10.39	11.76
Ni-O Distances (Å)	2.18, 1.92, 1.87	1.92, 2.17, 1.89	1.90, 2.05, 1.99	2.18, 2.03, 1.99
Ni-O-Ni_B Angle (°) (Ni_B)	146.9, 146.8(Ni_2)	145.6 (Ni_3)	147.6, 148.0 (Ni_4)	147.6 (Ni_1)
**1/2 added electrons per Ni**				
Magnetic Moment (μ_B)	0.80	-0.80	1.63	-1.63
Octahedral Volume (Å^3)	10.47	10.47	11.41	11.41
Ni-O Distances (Å)	2.18, 1.93, 1.87	1.93, 2.18, 1.87	2.04, 2.08, 2.03	2.08, 2.04, 2.03
Ni-O-Ni_B Angle (°) (Ni_B)	145.5(Ni_2)	144.6 (Ni_3)	145.4(Ni_4)	144.6 (Ni_1)
**3/4 added electrons per Ni**				
Magnetic Moment (μ_B)	0.75	-1.61	1.62	-1.62
Octahedral Volume (Å^3)	10.24	11.50	11.35	11.40
Ni-O Distances (Å)	2.09, 1.97, 1.88	2.05, 2.17, 1.96	2.06, 2.10, 1.98	2.06, 2.04, 2.04
Ni-O-Ni_B Angle (°) (Ni_B)	143.6, 144.4(Ni_2)	141.4 (Ni_3)	144.5, 143.6 (Ni_4)	142.8 (Ni_1)
**1 added electron per Ni**				
Magnetic Moment (μ_B)	1.61	-1.61	1.61	-1.61
Octahedral Volume (Å^3)	11.31	11.31	11.31	11.31
Ni-O Distances (Å)	2.09, 2.06, 1.98	2.06, 2.09, 1.98	2.06, 2.09, 1.98	2.09,2.06,1.98
Ni-O-Ni_B Angle (°) (Ni_B)	142.5(Ni_2)	139.3 (Ni_3)	142.5 (Ni_4)	139.3 (Ni_1)
**1 added electron per Ni (Lattice Parameters Relaxed, Supplementary Fig. 5a, third panel)**				
Magnetic Moment (μ_B)	1.66	-1.66	1.66	-1.66
Octahedral Volume (Å^3)	13.78	13.78	13.78	13.78
Ni-O Distances (Å)	2.27, 2.11, 2.15	2.11, 2.27, 2.15	2.11, 2.27, 2.15	2.27, 2.11, 2.15
Ni-O-Ni_B Angle (°) (Ni_B)	140.7(Ni_2)	136.2 (Ni_3)	140.7 (Ni_4)	136.2 (Ni_1)

Supplementary Note 3: Adaptive Synaptic Plasticity Learning

In the Spiking Neural Network (SNN) simulations for digit recognition, we use the Leaky-Integrate-and-Fire (LIF) model[13,14] to simulate the membrane potential V of a neuron as

$$\tau \frac{dV}{dt} = (E_{\text{rest}} - V) + g_{\text{e}} * (E_{\text{exc}} - V) + g_{\text{i}} * (E_{\text{inh}} - V)$$

where E_{rest} is the resting membrane potential (-65 mV), E_{exc} (0 mV) and E_{inh} (-100mV) are the equilibrium potentials of excitatory and inhibitory synapses, τ is the time constant (100 ms) and g_{e} and g_{i} are the conductances of excitatory and inhibitory synapses respectively. The LIF model causes V to increase when pre-synaptic spikes are received and to otherwise decay exponentially. The post-neuron fires when V crosses the membrane threshold V_{thresh} (-52 mV) and THEN its membrane potential is reset to V_{rst} (-65 mV). After each firing event, a refractory period (5 ms) ensues during which the post-neuron is inhibited from firing even if additional input spikes arrive.

Synapses are modeled by conductance changes[13,14] wherein the conductance increases by the synaptic weight, w, only upon the arrival of pre-neuronal spike. Otherwise, the conductance continues to decay exponentially. The dynamics of both inhibitory and excitatory conductance are simulated as

$$\tau_{\text{e}} \frac{dg_{\text{e}}}{dt} = -g_{\text{e}}, \qquad \tau_{\text{i}} \frac{dg_{\text{i}}}{dt} = -g_{\text{i}}$$

where τ_{e} (1ms) or τ_{i} (2ms) are the time constants for the excitatory or inhibitory post-synaptic potential.

As discussed in Supplementary Fig. 9, homeostasis is used to prevent a single neuron from dominating the spiking pattern. Specifically, each excitatory neuron's membrane threshold is not only determined by V_{thresh} but by $V_{\text{thresh}} + \theta$, where θ is increased each time the neuron fires and then decays exponentially at an extremely slow rate. We use $\theta = 0.1$ and a very high decay time constant of 10^8 ms in our simulations.

Each input image is presented for 350 ms. There is resting period of 150 ms before presenting a new input to allow all neuronal parameters to decay to the reset values (except for the adaptive membrane threshold, $V_{\text{thresh}} + \theta$). We note to the reader that we use identical parameters for neuron and synapse models, input encoding and input image presentation time as Diehl & Cook[14] for fair comparison of our ASP learning with standard STDP learning (Fig. 3b, Supplementary Fig. 10). The standard STDP learning model is implemented using the power law weight dependent rule[14,15].

ASP: Learning rules

We examine the mathematical formulations for ASP to understand how the temporal dynamics dictate the plasticity that eventually enables the SNN to learn to forget as well as adapt to new patterns.

Recovery Phase

To improve simulation speed, the weight dynamics are computed using synaptic traces[16]. In ASP learning, the synapses keep track of three different kinds of traces corresponding to pre and post-synaptic neuron's spiking activity: a) Recent presynaptic trace (Pre_{rec}) that doesn't accumulate over time (only accounts for the most recent spike), b) Accumulative presynaptic (Pre_{acc}) trace that adds over time (accounts for the entire spike history of the presynaptic neuron for a given time period or epoch during which a particular pattern is presented to the SNN), c) Postsynaptic trace ($Post$) that accumulates over time based on the postsynaptic neuron's spiking activity. Each of the traces is evaluated as follows wherein the trace is increased when a spiking activity is observed, otherwise it decays exponentially:

$$Pre_{\text{rec}}(t) = \exp\left(-\frac{Pre_{\text{rec}}}{\tau_{\text{rec}}}\right);\ Pre_{\text{rec}} = 1 \text{ when } t_{\text{pre}} \text{ occurs} \qquad (1)$$

$$Pre_{\text{acc}}(t) = \exp\left(-\frac{Pre_{\text{acc}}}{\tau_{\text{acc}}}\right);\ Pre_{\text{acc}} += 1 \text{ when } t_{\text{pre}} \text{ occurs} \quad (2)$$

$$Post(t) = \exp\left(-\frac{Post}{\tau_{\text{post}}}\right);\ Pre_{acc} += 1 \text{ when } t_{\text{post}} \text{ occurs} \quad (3)$$

Now, the time constant for decay of the accumulative pre-trace (Pre_{acc}) has to be larger than that of the recent pre-trace (Pre_{rec}) so that spike history can be appropriately added. In our simulations, $\tau_{\text{acc}} = 10\tau_{\text{rec}}, \tau_{\text{post}} = 2\tau_{\text{acc}}$. We adopt a modified version of the power-law weight dependent STDP model[13,14] to obtain the weight changes during the recovery phase (i.e. in presence of input stimulus) of ASP. When a postsynaptic spike arrives at the synapse, the weight change Δw is calculated based on the presynaptic trace (Pre_{rec}, Pre_{acc})

$$\Delta w = \eta(t)(Pre_{\text{rec}} - offset) - \frac{k_{\text{const}}}{2^{Pre_{\text{acc}}}} \quad (4)$$

where $\eta(t)$ is a time dependent learning rate that is inversely proportional to the post-synaptic trace value ($Post(t)$ from Eqn. 3) at a given time instant. As the post-synaptic neuron in the excitatory layer starts spiking for a given input, the learning rate will decrease. This will ensure that a particular neuron retains and stably learns a particular input pattern. It also prevents the neuron from quickly adapting to a new pattern (or catastrophic forgetting). The *offset* ensures that the presynaptic neurons that rarely lead to firing of the postsynaptic neuron will become more and more disconnected (or the synaptic weight values will depress). In case of digit inputs, the black (or off) pixel region for a particular digit will become disconnected resulting in lowering of synaptic weight values corresponding to the pre-neurons in the lower pixel intensity region. In Eqn. 4, the first part represents the weight change (potentiation or depression) based on the most recent pre-synaptic spike (as with STDP). However, as seen earlier, erasure of memory traces is prominent with STDP as in its simplest form any pre/post spike pair will modify the synapse. Besides precise spike timings that identify the correlation between input patterns, learning rule should incorporate the significance of the inputs to modulate the weights. As the inputs are continually changed, an SNN (with fixed resources or size) should gradually forget obsolete data while retaining important information. Thus, input based significance driven learning would enable the SNN to learn in a stable-plastic manner in a dynamic environment.

The second part of Eqn. 4 quantifies the dependence of the weight change on the significance of the input pre-neuron. We define an input neuron to be significant if it has more frequent spikes. In that case, Pre_{acc} value will be high that would eventually make the second term in Eqn. 4 less dominant for determining the final weight update. Thus, for more frequent input spikes at the pre-neuron, the weight update will be more prominent. Hence, the learning rule encompasses significance of the inputs with standard synaptic plasticity. It can be deduced that the prominent weights will essentially encode the features that are common to different input classes as the pre-neurons across those common feature regions in the input image will have higher firing activity. This eventually helps the SNN to learn more common features across different input patterns to obtain more generic representation of the data.

Decay Phase

The decay phase in ASP learning is activated in the absence of input stimulus i.e. when no spiking activity is observed at the synaptic terminals connecting pre and post neuron. It involves the forgetting of the weights for insignificant information to enable the SNN to learn new data without catastrophic forgetting or overlap of representations. As discussed earlier, the weights undergo an exponential decay towards a baseline value as

$$\tau_{\text{leak}} \frac{dw}{dt} = -\alpha w \quad (5)$$

where α is a decay constant and τ_{leak} is the time constant of decay. τ_{leak} is a time dependent quantity that is proportional to the post-synaptic trace value (*Post*(*t*) from Eqn. 3) and the membrane threshold value ($V_{\text{thresh}} + \theta$ obtained from homeostasis behavior) at a given time instant. Now, it is desirable that the weights that have learnt a pattern should leak less in order to retain the learnt information. A neuron that has learnt a particular pattern will have a higher spiking activity (or higher post trace value *Post*(*t*)) that will increase the time constant of decay, τ_{leak}. Higher τ_{leak} causes the weight to forget or leak less. *Post*(*t*) will be higher for an excitatory neuron that has learnt an input pattern that is recent and presented latest to the network. The overall leak rate can be defined as $\alpha/\tau_{\text{leak}}$ that decreases with increasing τ_{leak}.

While *Post*(*t*) is indicative of how recent and latest the input pattern is, it does not account for the significance of the input pattern. We define significance in terms of number of times a particular pattern has been presented to an SNN. The membrane threshold (obtained from homeostasis) of a post-neuron is representative of the significance of the input pattern. A neuron that has learnt a given pattern will spike more when that pattern is presented several times to the network. An excitatory layer neuron's membrane threshold will be high only when it is firing more. Higher membrane threshold implies that the corresponding excitatory layer neuron has learnt a significant pattern. Hence, the SNN learns to forget insignificant older information while trying to retain more recent and significant, yet old, data using ASP.

A key aspect to note here is that the weight leak in the decay phase is based only on the post-neuron's spiking activity (and membrane threshold). All weights connected to a post-neuron in the excitatory layer will have the same decay time constant, τ_{leak} and hence show uniform leak dynamics during the decay phase. On the contrary, during recovery phase, the weight dynamics of each synapse will be different as it is determined by both the post and pre-neuronal spiking activity.

Supplementary Note 4: ASP-based learning to forget is compatible with other proposed neuromorphic device technologies

As discussed earlier, the dopant interaction with the perovskite lattice seen in experiment and studied by *ab initio* dynamical simulations enables habituation-based plasticity. This is key to the perovskite's forgetting capability that motivates the ASP learning. In recent years, non-volatile and/or filamentary switch device elements including spin-based and memristors to emulate the behavior of neural systems have been proposed[17-25]. While our correlated perovskite can emulate forgetting similar to the animal world, other non-volatile devices can be made to forget or leak their conductance by applying electrical pulses. Our proposed ASP can therefore be synergistic with those devices as well. Thus, ASP can be incorporated with a broad range of programmable devices to construct robust self-adaptive artificial neural systems for dynamic environments.

Supplementary Note 5: AIMD Movie

Ab initio molecular dynamics simulations showing migration of proton in H-doped monoclinic SNO crystal at 300 K. The proton hops from one O atom to another neighboring O atom within the NiO_6 octahedron in a facile manner (see Fig. 2e for details on the activation barriers). The Ni, O, Sm and H are depicted as green, red, yellow, and blue spheres respectively. For the sake of clarity, only the hydrogen, and the Ni/O atoms belonging to the two NiO_6 octahedra closest to the hydrogen are shown as large spheres; the atoms far away from the hopping phenomena are depicted with small translucent spheres. Our AIMD simulations at 300 K at various H doping levels show that the SNO lattice monotonically expands with addition of hydrogen approaching lattice expansion of ~5% for 1 H per unit cell of SNO.

Supplementary References

1 Zhou, Y. *et al.* Strongly correlated perovskite fuel cells. *Nature* **534**, 231-234 (2016).

2 Bruno, F. *et al.* Probing the metal-insulator transition in nickelates using soft x-ray absorption spectroscopy. *Appl. Phys. Lett.* **104**, 021920 (2014).

3 LeCun, Y., Bottou, L., Bengio, Y. & Haffner, P. Gradient-based learning applied to document recognition. *Proc. IEEE* **86**, 2278-2324 (1998).

4 Zhang, W. & Linden, D. J. The other side of the engram: experience-driven changes in neuronal intrinsic excitability. *Nat. Rev. Neurosci.* **4**, 885-900 (2003).

5 Cyr, A., Boukadoum, M. & Thériault, F. Operant conditioning: a minimal components requirement in artificial spiking neurons designed for bio-inspired robot's controller. *Front.Neurorobot.* **8**, 21 (2014).

6 Boukadoum, M. Habituation: a non-associative learning rule design for spiking neurons and an autonomous mobile robots implementation. *Bioinspir. Biomim.* **8**, 016007 (2013).

7 Cyr, A. & Boukadoum, M. Classical conditioning in different temporal constraints: an STDP learning rule for robots controlled by spiking neural networks. *Adapt. Behav.* **20**, 257-272 (2012).

8 Grossberg, S. & Swaminathan, G. A laminar cortical model for 3D perception of slanted and curved surfaces and of 2D images: development, attention, and bistability. *Vision Res.* **44**, 1147-1187 (2004).

9 Grossberg, S. Towards a unified theory of neocortex: laminar cortical circuits for vision and cognition. *Prog. Brain Res.* **165**, 79-104 (2007).

10 Park, H., Millis, A. J. & Marianetti, C. A. Total energy calculations using DFT+ DMFT: Computing the pressure phase diagram of the rare earth nickelates. *Phys. Rev. B* **89**, 245133 (2014).

11 Medarde, M. *et al.* Charge disproportionation in $RNiO_3$ perovskites (R= rare earth) from high-resolution x-ray absorption spectroscopy. *Phys. Rev. B* **80**, 245105 (2009).

12 Alonso, J. A., Martinez-Lope, M. J., Casais, M. T., Aranda, M. A. & Fernandez-Diaz, M. T. Metal-insulator transitions, structural and microstructural evolution of $RNiO_3$ (R= Sm, Eu, Gd, Dy, Ho, Y) perovskites: Evidence for room-temperature charge disproportionation in monoclinic $HoNiO_3$ and $YNiO_3$. *J. Am. Chem. Soc.* **121**, 4754-4762 (1999).

13 Song, S., Miller, K. D. & Abbott, L. F. Competitive Hebbian learning through spike-timing-dependent synaptic plasticity. *Nat. neurosci.* **3**, 919-926 (2000).

14 Diehl, P. U. & Cook, M. Unsupervised learning of digit recognition using spike-timing-dependent plasticity. *Front. Comput. Neurosci.* **9**, 99 (2015).

15 Querlioz, D., Bichler, O., Dollfus, P. & Gamrat, C. Immunity to device variations in a spiking neural network with memristive nanodevices. *IEEE Trans. Nanotechnol.* **12**, 288-295 (2013).

16 Morrison, A., Aertsen, A. & Diesmann, M. Spike-timing-dependent plasticity in balanced random networks. *Neural Comput.* **19**, 1437-1467 (2007).

17 Prezioso, M. *et al.* Training and operation of an integrated neuromorphic network based on metal-oxide memristors. *Nature* **521**, 61-64 (2015).

18 Locatelli, N., Cros, V. & Grollier, J. Spin-torque building blocks. *Nat. Mater.* **13**, 11-20 (2014).

19 Tuma, T., Pantazi, A., Le Gallo, M., Sebastian, A. & Eleftheriou, E. Stochastic phase-change neurons. *Nat. Nanotechnol.* **11**, 693-699 (2016).

20 Shi, J., Ha, S. D., Zhou, Y., Schoofs, F. & Ramanathan, S. A correlated nickelate synaptic transistor. *Nat. Commun.* **4**, 2676 (2013).17

21 Yang, J. J., Strukov, D. B. & Stewart, D. R. Memristive devices for computing. *Nat. Nanotechnol.* **8**, 13-24 (2013).

22 Pickett, M. D., Medeiros-Ribeiro, G. & Williams, R. S. A scalable neuristor built with Mott memristors. *Nat. Mater.* **12**, 114-117 (2013).

23 Ohno, T. et al. Short-term plasticity and long-term potentiation mimicked in single inorganic synapses. *Nat. Mater.* **10**, 591-595 (2011).

24 Fong, X. et al. Spin-transfer torque devices for logic and memory: Prospects and perspectives. *IEEE Trans. Comput-Aided Des.* **35**, 1-22 (2016).
25 Wong, H.-S. P. *et al.* Metal-oxide RRAM. *Proc. IEEE* **100**, 1951-1970 (2012).
